# Change of Location for Health

**Published:** 1883-01

**Authors:** 


### CHANGE OF LOCATION FOR HEALTH.

CHANGE of location for invalids is an important curative element of itself, without reference to change of climate. As a rule persons who have contracted diseases while residing near the sea, receive the most marked benefit by changing their residence to a location "inland," and beyond the influence of the salt air; on the contrary, people who reside inland, when health fails, are benefited by changing their residence to some point near the sea. This is most marked in diseases of the throat, and in less degree in diseases of the chest. But then in almost all diseases benefit is derived from a change of location, whether it be a point more or one less salubrious, than the place of departure. Now we believe that climatic changes have, ordinarily, very little to do with it, and that the improved condition is almost wholly due to pleasurable emotions excited in the mind by the new scenes and incidents which interest it and keep it occupied. This fact is an illustration of the power of the mind over the body. We see this power displayed in every act of our lives. What is called "moral force" is supreme, properly directed it would eliminate more than half the ills of life. But let the mind become chained to an idea, and all the skill of medical men is often powerless against it. Let a patient be thoroughly convinced that he is the victim of a fatal disease, and if in due time he is not convinced to the contrary, he may bring the disease upon himself or die from fears that—too late—have proved groundless. We would not discourage the use of medicines where their employment is plainly indicated; but there are thousands who are suffering from ailments that can not well be defined, and which affect both mind and body. The remedy is change. Go! no matter where. Do! no matter what, so long as it is worthy. In our opinion, the great mass of semi-invalids simply need a thorough shaking up. Give the Immortal Mind something better and nobler to do than to be constantly occupied with troubles that are imaginary.

